# Methodological Aspects of Slow-Paced Breathing in Healthy Young Individuals with Optimal and Suboptimal Spirometric Indices

**DOI:** 10.3390/life16060970

**Published:** 2026-06-09

**Authors:** Liliya Poskotinova, Elena Andreeva

**Affiliations:** 1 N. Laverov Federal Center for Integrated Arctic Research of the Ural Branch of the Russian Academy of Sciences, Arkhangelsk 163020, Russia; 2 Department of Family Medicine, Northern State Medical University, Arkhangelsk 163001, Russia

**Keywords:** slow-paced breathing, spirometry, heart rate variability, young adults

## Abstract

Background: The methodology of slow-paced breathing (SPB) to optimize cardiorespiratory function requires further refinement. The aim of this study was to identify the methodological ranges of resonance breathing frequencies during SPB in healthy young adults with optimal and suboptimal spirometric indices. Methods: Twenty-eight healthy Indian students living in the Arctic Russian region (age 21–24 years; 20 males, 8 females) underwent spirometry and 2 min HRV recordings at 6 time points: baseline (spontaneous breathing) and SPB periods of 9–13 s with biofeedback. Results: Participants were divided into Group 1 (FEV1 ≥ 80% predicted, n = 13) and Group 2 (FEV1 < 80% predicted, n = 15). At baseline, Group 2 showed reduced overall HRV, lower vagal activity, a higher stress index and lower baroreflex power (by LFmx) vs. Group 1. During SPB, Group 1 exhibited more significant vagal enhancement, peaking at 9–10 s breathing periods. Group 2 showed delayed, wave-like responses with peak effects at 9 s and/or 12–13 s. Conclusions: SPB frequency depends on the level of FEV1: 9–10 s periods in individuals with FEV1 ≥ 80% predicted, and 9 s and/or 12–13 s periods (delayed effect) in those with FEV1 < 80% predicted, which may be due to cardiorespiratory strain under uncomfortable climatic conditions.

## 1. Introduction

Slow-paced breathing (SPB) has emerged as a powerful non-pharmacological intervention in physiology and medicine, promoting autonomic balance, reducing stress, and improving cardiovascular and neurocognitive functioning through targeted modulation of breathing rhythms [1]. This technique exploits the interaction between respiration and heart rate variability (HRV), fostering coherence of cardiorespiratory oscillations that supports overall homeostasis, as demonstrated by comprehensive reviews linking SPB to enhanced vagal tone and improved emotion regulation [1]. A breathing frequency of 0.1 Hz (equivalent to 6 breaths per minute) is the most commonly applied rate in SPB protocols because it aligns with peak vagal activity and baroreflex sensitivity [1]. Studies indicate that this resonance frequency optimizes low-frequency HRV power, augmenting parasympathetic outflow and cardiovascular stability, which underlies its efficacy in clinical groups ranging from patients with hypertension [2] to adolescents with intellectual disability [3]. This frequency harnesses the natural resonance of the cardiovascular system, whereby slow inhalation–exhalation cycles synchronize with Mayer waves, leading to measurable increases in HRV indices such as RMSSD and high-frequency power [4]. At the same time, SPB-induced changes in cerebral blood flow also involve limbic brain regions, suggesting the optimization not only of cardiovascular function but also of brain–heart interactions [1].

However, several studies indicate that there is no single universal resonance breathing frequency in humans. In the study by Pagaduan et al. [4], cardiorespiratory and hemodynamic effects at different breathing frequencies over 2 min trials were examined in healthy men. It was shown that both at the individual resonance frequency and at breathing frequencies exceeding the resonance frequency by more than one breath per minute, similar cardio- and hemodynamic changes occurred [4], while baroreflex activity remained stable. Overall, effective slow-paced breathing appears to be associated with a range of resonance breathing periods between approximately 8 and 13 s, which may reflect alterations in psycho-emotional regulation as well as cardiovascular dysfunction [5]. In general, current researchers emphasize the growing prevalence of breathing disturbances in contemporary youth and the use of slow breathing techniques for correcting not only cardiopulmonary, but also neuropsychiatric disorders [3,6,7,8]. It is emphasized that the resonance frequency is individual and usually lies between 4.5 and 7 breaths per minute, with 6 breaths per minute being only the most commonly used approximation [9]. Nevertheless, the methodological aspects of selecting an individual resonance breathing frequency during SPB in healthy individuals with suboptimal spirometric indices, indicative of respiratory dysfunction, remain poorly defined.

Suboptimal health status has been recognized as a distinct condition in modern populations, in which physiological status remains uncertain for a prolonged period of time [10,11]. However, reduced spirometric parameters are not considered within this framework of suboptimal health, highlighting the need to accumulate data on “non-optimal” (or suboptimal) spirometric indices as potentially valuable predictors of health disturbances, particularly in young people.

Suboptimal spirometric parameters can clarify the risk of impaired adaptation of the bronchopulmonary system to the challenges of the living environment. Early subclinical respiratory changes in patients may manifest as preserved ratio impaired spirometry (PRISm) [12,13]. This pattern is characterized by a reduced forced expiratory volume in 1 s (FEV1, % predicted) below 80%, combined with a preserved ratio of FEV1 to forced vital capacity (FEV1/FVC) of 0.7 or higher. In adults with PRISm, subsequent trajectories include both progression to chronic obstructive pulmonary disease and favorable outcomes with normalization of spirometric parameters [13,14]. Such divergent trajectories (maintenance of health, development of lung disease, or even death) largely depend on lifestyle, the frequency of respiratory infections, comorbidities, and smoking [14], as well as discomfort related to environmental conditions [13].

An important marker of suboptimal respiratory function is a reduced FEV1, which is a robust indicator of overall human health but is still insufficiently used as a biomarker of quality of life in young adults [15,16]. The question of the FEV1 threshold below which disease risk should be considered remains under debate. Although an FEV1 between 80 and 100% of predicted is often regarded as “normal”, a more relevant question is whether this level represents the individual’s maximal attainable function [16]. The author reasonably calls for further research to identify which aspects of low FEV1 or FVC values warrant intervention even when they fall within the conventional normal range but remain below the population average [16]. In the study by Madeline F. Cannon et al., it was demonstrated that a single FEV1 measurement in individuals working under adverse conditions (firefighters) can predict mortality over the subsequent two decades, even when FEV1 lies within the normal range, and that each 10% decrement in FEV1 within the range of 109–80% predicted is associated with a progressive increase in mortality [17]. A state with FEV1 below 80% but above 70% in non-COPD, non-asthmatic, non-smoking healthy subjects is considered a risk condition for future pulmonary impairment, as it is associated with polymorphisms in specific genes [18].

Therefore, it is highly relevant to investigate the impact of suboptimal spirometric levels, with particular emphasis on reduced FEV1 (<80% of predicted value), on the reactivity of regulatory systems in healthy individuals without clinically confirmed disease. Identifying breathing frequencies that correspond to individual resonance points is crucial, because standard 0.1 Hz protocols are often ineffective, necessitating personalized spirometric and breathing assessments to avoid inefficacy or undesirable cardiorespiratory load. The search for optimal breathing regimes also remains important in the context of physical rehabilitation in patients with clinically significant obstructive lung diseases [19]. Living in climatically uncomfortable conditions provides a convenient experimental model to examine possible changes in respiratory function in healthy individuals, thereby allowing different cardiorespiratory and autonomic nervous effects of SPB to be demonstrated. Our previous studies showed that in students arriving from India to study in the Arctic Russian region, the biofeedback-induced increase in overall heart rate variability (HRV), achieved by individual adjustment of breathing rate and depth, differed from that observed in permanent Arctic residents [20,21]. It is assumed that differences in cardiovascular and neurophysiological effects of biofeedback training may be related to the specific spirometric status of the Indian students.

Thus, involving young individuals living in adverse climatic conditions appears to be an effective experimental approach for comparing groups with optimal and suboptimal spirometric parameters. The aim of the present study was to identify the methodological ranges of resonance breathing frequencies during SPB associated with the maximal activation of baroreflex activity and vagal heart rate modulation in healthy adults with optimal and suboptimal spirometric status, with a specific focus on reduced FEV1 (<80% of predicted value).

## 2. Materials and Methods

### 2.1. Study Participants

This study is presented as a pilot study. It involved 28 Indian medical students (aged 21–24 years; 20 males, 8 females) who had resided in the Russian Federation for 4 years while studying at a medical university in Arkhangelsk (the Russian Arctic region). The sample was formed based on the principle of “convenience sampling” [22]. Informed voluntary consent was obtained from all students. The protocol of this study conformed to the Declaration of Helsinki for human experimentation. For each participant, a questionnaire was completed indicating their height, health status, and blood pressure (to exclude arterial hypertension). The inclusion criteria were: residents of India who had arrived to study in the Arctic region, age 21–24 years, and the presence of voluntary informed consent for the examination. The exclusion criteria were: chronic pathology of the cardiorespiratory system, allergic diseases, endocrine-metabolic disorders, and acute infectious diseases at the time of the study. All participants were non-smokers.

### 2.2. Study Procedures

#### 2.2.1. Spirometric Examination

Respiratory parameters such as forced expiratory volume in 1 s (FEV1, L), forced expiratory volume in 6 s (FEV6, L), FEV1% and FEV6% of predicted and FEV1/FEV6 ratio were measured with the “Vitalograph COPD-6” (Manufacturer: Vitalograph, Ennis, Ireland), a handheld respiratory monitor, designed for lung function testing in adults and children within home and professional healthcare environments. Participants performed three, 6 s maximal expiratory maneuvers; the highest FEV1 and FEV6 values, and their ratio, from the three measurements were used for analysis. The device is intended to be operated by the patient under the supervision of a healthcare provider. Participants were initially stratified according to their pre-bronchodilator FEV1 value: Group 1—normal FEV1 (80% and above of predicted) with a normal FEV1/FEV6 ratio (>0.7); Group 2—low FEV1 (below 80% of predicted) with a normal FEV1/FEV6 ratio (>0.7).

#### 2.2.2. Heart Rate Variability Analysis During SPB Dynamics

Cardiorhythmogram (inter-beat rhythmogram) registration according to the first standard ECG lead in the sitting position was performed on participants using the “Varicard” device (“Ramena”, Ryazan, Russia) at several time points (T): with free breathing (T1—baseline), followed by T2 to T6 with breathing periods from 9 to 13 s (9 s, 10 s, 11 s, 12 s, and 13 s). A total of 6 time points were recorded, each with 2 min of inter-beat rhythmogram registration. Slow-paced breathing was performed with biological feedback using a signal window on a laptop monitor with the “Varicard” device software. In the signal window, at the beginning of each breathing cycle, the word “Inhale” appeared, signaling the participant to inhale. Next, a colored bar was displayed to the participant in the signal window, indicating the continuation of the breathing cycle, exhalation, and completion of the breathing cycle. The reappearance of the word “Inhale” indicated repetition of the breathing cycle. The following HRV parameters were used for analysis [23,24]: heart rate (HR, bpm); maximum difference between maximum and minimum NN interval values (MxDMn, ms); ratio between maximum and minimum NN interval values (MxRMn); root mean square of successive differences of NN intervals (RMSSD, ms); percentage of consecutive NN intervals that differ by more than 50 ms (pNN50, %); standard deviation of NN intervals (SDNN, ms); stress index by Baevsky (SI, units) [25,26]; spectral total power of HRV (TP, 1000×, ms^2^); maximum of spectral power in low-frequency band of HRV or peak LF power density (LFmx, ms^2^/Hz); the period of peak LF power density (LFmx period, sec); high-frequency spectral power (HF, %); low-frequency spectral power (LF, %). Considering the ultrashort inter-beat rhythmogram recordings (less than 5 min), assessment of very low frequency HRV components was not performed.

### 2.3. Statistical Analysis

Statistical analyses were performed using STATISTICA software v. 13.0 (StatSoft, Inc., Tulsa, OK, USA). As the distribution of values in the samples did not follow a normal distribution (Shapiro–Wilk test), statistical processing was carried out using non-parametric methods. Quantitative parameters are presented as medians with ranges corresponding to the median (Me) with the 25th and 75th percentiles (p25 and p75). Comparisons of quantitative variables between independent groups (Group 1 and Group 2) were conducted using the Mann–Whitney U test (*p* < 0.05). Comparisons of quantitative variables between dependent groups (T1–T6) were conducted using the Friedman ANOVA by ranks test (*p* < 0.05). Dunn–Šidák post hoc tests (significance level *p* ≤ 0.010) were applied exclusively to pairwise comparisons of each time point from T2 to T6 against T1 (baseline), and no multiple comparisons were performed between time points T2–T6 themselves.

## 3. Results

The groups did not differ in age, height, or the male-to-female ratio (*p* > 0.05)—Table 1. In Group 2 individuals compared to Group 1 individuals, FEV1 (*p* = 0.004), FEV1% of predicted (*p* < 0.001), FEV6 (*p* = 0.019), and FEV6% of predicted (*p* = 0.017) values were significantly lower, but the FEV1/FEV6 ratio was statistically equal between groups (*p* = 0.088). Thus, in Group 2 individuals, absolute and % of predicted forced expiratory volumes (FEV1 and FEV6) were lower, but with a preserved FEV1/FEV6 ratio, indicating an adaptive reduction in the expiratory reserve without significant obstructive changes. Equality of groups in age, height, and the sex ratio excludes anthropometric artifacts, while the normal FEV1/FEV6 ratio (≥70–75%) indicates the absence of airflow slowing in the bronchial lumen. At time point 1 (T1—baseline), Group 2 individuals exhibited significantly reduced overall cardiogram variability compared to Group 1 (MxDMn, *p* = 0.004; MxRMn, *p* = 0.005; SDNN, *p* = 0.011), lower vagal activity by the pNN50 parameter (*p* = 0.037), and a higher stress index (SI, *p* = 0.010), reflecting a moderate shift in autonomic balance toward sympathicotonia.

Although no significant shift toward tachycardia was detected in Group 2 compared to Group 1 (HR, *p* = 0.253), this indicates the absence of pronounced hypoxic manifestations and increased oxygen demand in Group 2 individuals. Among spectral parameters, the power of the maximum spectral peak in the low-frequency range (LFmx) was notably significantly lower in Group 2 individuals (*p* = 0.011), reflecting reduced baroreflex activity power in Group 2. At the same time, the periods of the LF range maximum peak (LFmx period) were statistically equal between groups (*p* > 0.05).

**Figure 1:**
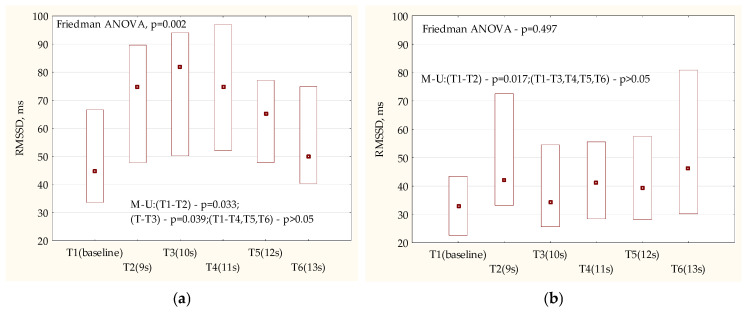
RMSSD dynamics (Me (p25; p75)) during slow-paced breathing with breathing cycles from T1 to T6 time points in Group 1 (**a**) and Group 2 (**b**).

During the SPB test at different breathing frequencies (with periods from 9 to 13 s), group differences were identified in the breathing frequencies corresponding to the greatest baroreflex activity and vagal activation. Thus, in Group 1 individuals, despite the lack of statistical significance in the Mann–Whitney test with multiple comparisons correction (more than *p* ≤ 0.010), there was nevertheless a systematic increase in the RMSSD during the test dynamics according to Friedman’s ANOVA test (*p* = 0.002), reaching a maximum at a breathing period of 9–10 s—Figure 1a. In Group 2 individuals, no significant increase in the RMSSD occurred during the test dynamics (Friedman ANOVA—*p* = 0.497), reflecting substantial variability in adaptive vagal heart rate regulation responses in Group 2—Figure 1b. In Group 2 individuals, the intergroup range of RMSSD was greatest at breathing periods of 9 and 13 s, which can be interpreted as a delayed response or training response in individuals with reduced lung function.

**Figure 2:**
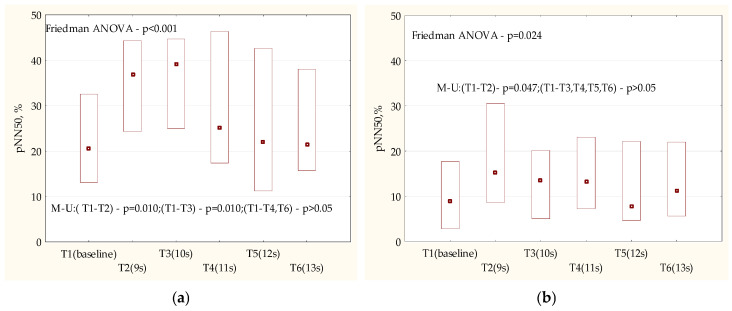
pNN50 dynamics (Me (p25; p75)) during slow-paced breathing with breathing cycles from T1 to T6 time points in Group 1 (**a**) and Group 2 (**b**).

The pNN50 parameter, which also characterizes vagal activity, showed more pronounced group differences when compared to the baseline (T1)—Figure 2. Thus, in Group 1 individuals, the pNN50 parameter increased significantly at timepoints T2 and T3 (periods of 9 and 10 s, respectively), whereas in Group 2 individuals, the significance level of the Mann–Whitney U test differences did not reach the required threshold for multiple comparisons of dependent groups compared to baseline values.

**Figure 3:**
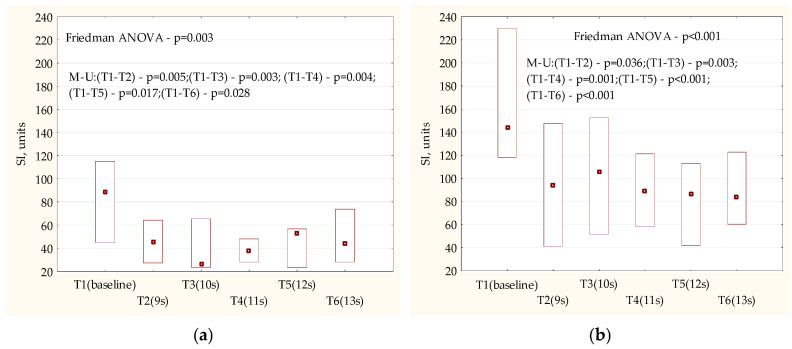
SI dynamics (Me (p25; p75)) during slow-paced breathing with breathing cycles from T1 to T6 time points in Group 1 (**a**) and Group 2 (**b**).

During SPB, sympathetic activity decreased significantly in both groups; however, the dynamics of SI reduction differed between the two groups. In Group 1 individuals, a significant SI reduction was observed from time points T2 to T4 (breathing periods from 9 to 11 s), reaching maximum reduction at a breathing period of 10 s (Figure 3a). In Group 2 individuals, significant SI reduction (at significance level *p* < 0.01) occurred at the 10 s period and continued through to 13 s—Figure 3b.

**Figure 4:**
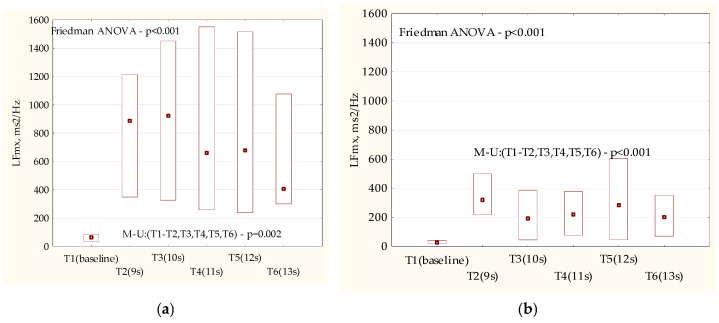
LFmx dynamics (Me (p25; p75)) during slow-paced breathing with breathing cycles from T1 to T6 time points in Group 1 (**a**) and Group 2 (**b**).

The LFmx parameter, reflecting baroreflex activity power, increased in Group 1 individuals to 890–918 ms^2^/Hz (*p* < 0.001) compared to the baseline (Figure 4a). The medians of this parameter reached maximum values at breathing periods of 9 and 10 s, although the interquartile ranges of this parameter remained consistently wide during breathing from 9 to 12 s, demonstrating intragroup individual differences in baroreflex activity dynamics during the test. In Group 2 individuals, although LFmx increased significantly during the SPB test dynamics (*p* < 0.001)—Figure 4b, the medians of this parameter were 2–3 times lower than in Group 1 individuals—reaching a maximum of 322 ms^2^/Hz at T2 (9 s period). After T2, the median LFmx remained stable with a significantly smaller interquartile spread (p25;p75) compared to Group 1 individuals, with some increase in the interquartile spread at the 12 s breathing period.

## 4. Discussion

During SPB performed at different breathing frequencies (with periods from 9 to 13 s), Group 1 individuals with FEV1 ≥ 80% predicted exhibited significant increases in vagal activity and decreases in sympathetic activity, particularly at a breathing period of 10 s (6 breaths per minute). The baroreflex response to respiratory stimulation, as determined by the LFmx parameter, was more powerful in Group 1 compared to Group 2, and most significant at breathing frequencies with periods of 9–10 s. Nevertheless, the intragroup range of this parameter reflected individual reactions expressed across all frequencies—from 9 to 12 s, and to a lesser extent at the 13 s period. In individuals with FEV1 below 80% compared to the group with a more optimal FEV1 level (≥80%), a lower variation range of cardiogram intervals was observed, along with less pronounced vagal influences and more pronounced sympathetic influences on the heart rhythm, as well as lower spectral power of the dominant low-frequency spectral component (LFmx), reflecting baroreflex activity.

In individuals with suboptimal spirometric values, changes in HRV parameters during respiratory tests exhibit a wave-like pattern, where increases in vagal influences and decreases in sympathetic activity occur most significantly first at a 9 s period and/or later closer to 13 s, which can be interpreted as a slower training response and a delayed cardiorespiratory system reaction to respiratory tests. These changes in individuals with suboptimal spirometric values could indicate the low adaptive plasticity of the cardio-respiratory coupling mechanism, where the transition to slower breathing is initially possible at a more comfortable breathing frequency—not 6 breaths per minute, but somewhat faster (around 7 breaths per cycle).

Our data confirm that the increase in the LFmx parameter, reflecting baroreflex response power during SPB, in individuals with FEV1 ≥ 80% predicted occurs equally at adjacent breathing frequencies—both at a breathing period of 10 s (6 breaths per minute) and at 9 s (6.6–7 breaths per minute) [4]. In the article by researchers [27] was shown that the greatest enhancement of baroreflex activity, measured by HRV parameters, in healthy individuals occurs at the resonance breathing frequency. Our study also demonstrated a similar phenomenon: during SPB periods of 9–10 s in individuals with FEV1 predicted ≥80%, the greatest reduction in sympathetic activity and increase in the LFmx parameter occurred.

However, in individuals with suboptimal spirometric values, the ratios of vagal and sympathetic regulation parameters at different SPB frequencies differed, which is associated with weakly expressed baroreflex activity in these individuals. It is considered that the optimal resonance breathing frequency, associated with the maximum increase in vagal heart regulation while hyperventilation is controlled, is an SPB period of 10 s or 0.1 Hz [28]. However, our data demonstrate that in individuals with optimal external respiration function parameters, the greatest increase in vagal activity and baroreflex response power occurs in the 9–10 s range. This may suggest a potential influence of climatic factors on cardiorespiratory system adaptation processes in residents of southern regions residing in the Arctic. Our previous studies showed that after physical exercise in a cold environment, the dominant period of the low-frequency HRV maximum peak (LFmx) in young local Arctic region residents was in the range of 9.2–9.8 s. After physical exercise in a cold environment, a single biofeedback session was conducted to increase total HRV, the effect of which was achieved with enhanced LFmx power and a median LFmx period of 9.4 s, with an interquartile range of 8.1–10.9 s [29]. Thus, both local Arctic region residents and Indian residents living here have a wide range of breathing frequencies at which the maximum vagal influence on the heart rhythm is achieved, predominantly at periods of 9–10 s. This may be due to adaptive strain in the ventilatory mechanisms of the bronchopulmonary system under cold Arctic climate conditions, which may contribute to an increased risk of subclinical respiratory obstruction [19,30].

This study is presented as a pilot study and therefore has several limitations. The group consists predominantly of males; therefore, recruitment will continue in the future with an increased proportion of females to identify sex differences in HRV reactivity during SPB. The status of smoking was not taken into account when forming the sample, which could have influenced the study results. Spirometry was performed using a portable COPD-6 spirometer; however, this device can serve as a screening tool for detecting spirometric abnormalities, including PRISm [31,32]. For future comparative studies, a group of local young people—university students born and permanently residing in the Arctic region—will be formed A more representative sample will also allow, in the future, the differentiation of groups with regard to smoking status, which is important for identifying different spirometric patterns (for example, PRISm or subclinical respiratory obstruction). Additionally, a comparative study using diagnostic spirometry with a bronchodilator test will allow the evaluation of the effectiveness of this inexpensive and simple screening test not only for screening but also for diagnosing external respiration function disorders.

## 5. Conclusions

In young people with FEV1 ≥ 80% predicted, during the SPB test with breathing periods from 9 to 13 s, significant increases in vagal activity and decreases in sympathetic activity occur, particularly at SPB periods of 9–10 s. In individuals with suboptimal spirometric indices, mainly FEV1 < 80% predicted, increases in vagal activity and baroreflex response power during the SPB test dynamics are less pronounced than in individuals with FEV1 ≥ 80% predicted, with HRV parameter changes occurring most significantly at an SPB period of 9 s and/or subsequently at periods of 12–13 s. The obtained data have practical significance for the individualized selection of the optimal breathing frequency during slow-paced breathing, taking into account the presence or absence of suboptimal spirometric indices, especially in individuals with strain of the cardiorespiratory system under uncomfortable climatic conditions.

## Figures and Tables

**Table 1:** Cardiorespiratory parameters at T1 (baseline) in persons with FEV1 ≥ 80% predicted (Group 1) and with FEV1 < 80% predicted (Group 2)—Me (p25; 75).

Variables	Group 1 (n = 13)	Group 2 (n = 15)	*p* Mann- Whitney Test
Age, years	23.0 (22.0; 23.0)	22.0 (21.0; 23.0)	0.467
Male/Female	10/3	10/5	0.558
Height, cm	178.0 (166.0; 181.0)	169.0 (164.0; 174.0)	0.253
FEV1, L	3.8 (3.1; 4.1)	2.8 (2.6; 3.0)	0.004
FEV1, % predicted	84.0 (83.0; 92.0)	72.0 (66.0; 77.0)	<0.001
FEV6, L	4.3 (3.7; 4.8)	3.3 (3.1; 3.9)	0.019
FEV6, % predicted	89.0 (84.0; 90.0)	80.0 (69.0; 85.0)	0.017
FEV1/FEV6, %	0.9 (0.8; 0.9)	0.8 (0.7; 0.9)	0.088
HR, bpm	75.4 (71.9; 91.4)	81.0 (77.6; 86.3)	0.253
MxDMn, ms	277.5 (255.8; 377.5)	202.0 (173.0; 245.0)	0.004
MxRMn	1.4 (1.4;1.5)	1.3 (1.3; 1.4)	0.005
RMSSD, ms	44.7 (33.7; 66.6)	33.1 (22.6; 43.5)	0.169
pNN50, %	20.7 (13.1; 32.6)	9.0 (2.8; 17.5)	0.037
SDNN, ms	58.8 (47.4; 83.2)	40.2 (34.2; 47.4)	0.011
SI, units	88.1 (44.9; 115.1)	143.6 (118.3; 230.0)	0.010
TP, 1000×, ms^2^	3.68 (2.37; 6.08)	2.06 (1.36; 4.40)	0.072
LFmx, mc2/Hz	68.1 (37.0; 85.2)	27.0 (21.3; 41.3)	0.011
LFmx period, s	12.3 (9.8;15.1)	13.5 (9.4; 19.3)	0.892
HF, %	29.3 (27.3; 34.3)	26.8 (17.8; 43.1)	0.495
LF, %	53.4 (45.8; 62.7)	42.0 (31.2; 61.0)	0.118

## Data Availability

No new data were created or analyzed in this study. Data sharing is not applicable to this article.

## Abbreviations

The following abbreviations are used in this manuscript:

FEV	Forced expiratory volume
HR	Heart rate
HRV	Heart rate variability
HF	High frequency band of heart rate variability
LF	Low frequency band of heart rate variability
LFmx	Low frequency band of heart rate variability, the peak LF power density
LFmx period	Low frequency band of heart rate variability the period of peak LF power density
MxDMn	Maximum difference between maximum and minimum NN interval values
MxRMn	Ratio between maximum and minimum NN interval values
pNN50	Percentage of consecutive NN intervals that differ by more than 50 ms
RMSSD	Root mean square of NN interval of successive differences
SDNN	Standard deviation of NN intervals
SI	Stress index by Baesky
SPB	Slow-paced breathing
TP	Total power (spectral total power of heart rate variability)
